# Factors Influencing Mitigation of Risk of Waterborne Disease in Vietnam Among Small Scale Integrated Livestock Farmers

**DOI:** 10.3389/fvets.2018.00154

**Published:** 2018-07-09

**Authors:** David C. Hall, Quynh B. Le

**Affiliations:** Department of Ecosystem and Public Health, Faculty of Veterinary Medicine, University of Calgary, Calgary, AB, Canada

**Keywords:** water public health, biodiversity conservation, poverty alleviation, emerging infectious disease, agricultural policy

## Abstract

The integrated livestock, crops, and fish (VAC) model of integrated small scale agriculture has been important to economic and ecological sustainability in Vietnam for many centuries. Recently, emerging waterborne diseases including avian influenza as well as the potential for zoonotic disease arising from small scale farms have jeopardized the VAC model. In order to promote mitigation of the risk of waterborne and other diseases in the VAC system, there needs to be recognition of the significant predictors of such behavior, particularly with respect to water sources including well and rain water. We report primarily quantitative results of research generated from 300 farms in each of North and South Vietnam that indicate the small scale farmers who are more likely to engage in mitigation of waterborne disease are those who raise pigs, perceive themselves to be more at risk of HPAI infection from well water, report they are good livestock managers, value the advice of health care workers, and where a female household member is the decision maker for family health. These results bear importance to water and health policy formulators in rural Vietnam. (JEL I130, I180, O130, Q180, Q570).

**JEL CLASSIFICATIONS:**

I130: Health and economic development
I180: Public health
O130: Economic Development: Agriculture; Environment
Q180: Agricultural policy; Food policy
Q570: Ecological economics: biodiversity conservation

## Introduction

Agricultural production in Vietnam remains an integral part of the Southeast Asian country's economy accounting for 18.4% of GDP in 2013 (1). However, more than 80% of Vietnamese farmers are small scale producers (2), incorporating some form of livestock and directly or indirectly dependent on agriculture (3, 4). A small scale integrated farming model particularly popular in Vietnam is the VAC mode (*Vuon* = Garden, *Ao* = Pond, and *Chuong* = Livestock housing), incorporating crops such as rice, fish, and livestock including poultry (5).

For small scale farmers in Vietnam, the transmission of water related zoonotic diseases (WRZD) due to poor farm management and lack of access to health and veterinary services has been a major constraint to containing and reducing risk of zoonotic disease (6–10). Part of the problem has been lack of awareness and understanding of basic public health including risk factors for zoonotic disease such as contamination of water sources by livestock. This seems to be especially so for small scale integrated farmers (6, 11–14).

Waterborne zoonotic pathogens of highest concern include *Cryptosporidium, Giardia, E. coli, Campylobacter, Salmonella, Leptospira*, and *Schistosoma* (15). Several studies from Asia have indicated varying degrees of awareness of these and other pathogens; highest perception of risk exists among high risk groups including agricultural workers (16–20). Other studies have found that agricultural workers perceive water contaminated with animal waste to pose a human health risk (21, 22), but that this is an unavoidable risk related to production (23).

We are not aware of any public health campaign in Vietnam to reduce exposure to these waterborne zoonotic pathogens. However, the public awareness campaigns since 2006 to help prevent and control avian influenza (24–26) have included advice to protect wells or ponds from fecal contamination by wild birds, and to avoid contamination of wells and ponds when destroying waste from sick and dead birds. The intention has been to limit the role of water to act as a reservoir for influenza viruses, as well as reduce risk of transmission of between species (including humans), rather than prevent direct infection via pond or well water which is rare for H5N1. Nevertheless, direct infection via water has been reported (27, 28) and risk increases with bathing in communal ponds or working in contaminated fish ponds, for example, both of which are common activities in rural Vietnam.

We propose that there is a strong association between Vietnamese small scale farmers' perception of risk of disease of waterborne origin and engagement in mitigating actions related to protection of water sources from contamination. This paper reports on the primarily quantitative results (qualitative results are reported in a separate publication) of participatory research with small scale farmers in two provinces of Vietnam in which we tested this hypothesis. Several theories and models of risk perception and motivation for mitigation have emerged since the mid-20th century including the Health Belief Model (HBM) (29) which proposes people do or do not respond to perceptions and beliefs of risk including susceptibility and outcome. Other popular risk perception and motivation models include the Protection Motivation Theory (30) and the psychometric paradigm models of Starr (31) and Fischhoff et al. (32). As a theoretical basis for our hypothesis, we referred to the HBM, in particular the premise that one's beliefs about the severity of exposure to a health risk hazard, impact of outcome, and barriers to change influence engagement in health promoting behavior.

## Methodology

Our research was conducted in collaboration with colleagues in Vietnam from academia (veterinary and human medicine, animal production, aquaculture, public health, and water researchers), government, and industry. Using a cross-sectional study design of randomly selected households, we identified two cohorts of 300 small scale farmers for our study in each of North and South Vietnam (Thai Binh and An Giang provinces respectively). The provinces were chosen because they are reported to have relatively large numbers of small scale farms in the north and south [ranked eighth and second respectively; Govt. Vietnam GSO, (33)] and for the practical reason that our Vietnamese collaborators were able to identify teams of reliable agricultural enumerators in those provinces. Communes in these provinces were identified by our Vietnamese colleagues as highly representative of typical agricultural communities in Vietnam. Small scale farmers were identified by commune leaders and included in our sample frame based on low income levels, farming activities that included crops and fish, raising of some livestock including 50 or fewer poultry, access to at least one of the main water sources of rural farmers (e.g., pond, river, well, and canal), and willingness to participate in the research. This profile also fits with the FAO definition of small scale farming in Vietnam and for poultry and mixed farms (34). Furthermore, this general agricultural profile is typical for the VAC model in Vietnam (5). A randomized selection of 300^1^ households in each province was then drawn from the sample frame; number of communes can vary but sampling was distributed as evenly as was practically possible. Both quantitative and qualitative methods were employed in the research using questionnaires, interviews, focus groups, and discussion and training sessions. Software tools included Excel, Stata 13, and NVivo 10. Ethics certification was secured by both the University of Calgary and the Hanoi School of Public Health for this research.

Questionnaires and interviews were implemented from July to September 2013 by trained Vietnamese university graduates with experience in these methods. Prior to commencing the questionnaire, farmers were asked to read (or it was read to them) and agree to an informed consent statement approved by ethics review committees of both Canadian and Vietnamese research institutions conducting the study. There were no refusals to provide informed consent. One questionnaire of 145 variables and one semi-structured (open ended) interview was conducted per household in Vietnamese with an individual who identified themselves as a decision maker in the household. The time spent by enumerators per household was about 90 min. Interviews were digitally recorded. An enumeration team of ten persons per province included an experienced team leader who doubled as a trainer, data gatherers who also collected water samples. Methods for qualitative analysis and interpretation including coding, thematic analysis, and interrelating themes using narrative passage followed those outlined by Creswell et al. (36) and Patton et al. (37). Quantitative data collected described agricultural, household, demographic, and economic variables. Binary and multinomial choice variables were also collected describing perceptions and preferences including likelihood of infection with H5N1 virus from water sources, value of health information from various informal sources including neighbors and media, self-evaluation of ability to raise livestock and crops, importance of hand washing, and impact of cost as a barrier to seeking health intervention assistance.

To engage farmers in a more participatory approach to our research while gathering data on water quality, farmers were trained in the use of basic color change presence/absence tests for their drinking water and interpretation of results. The expectation was this would assist in establishing dialogue with the visiting researchers as well as contribute to some ownership of the research being conducted on their farm. This training took place the same day as invigilation of the questionnaire. Farmers were asked to use the indicator test (a high sensitivity, low specificity beta-glucuronidase test) to test for the presence of coliforms in their drinking water and record the result within 24 h. A water sample was also taken from the drinking water source to be tested for pH, turbidity, and level of *E. coli* in a national microbiology laboratory in Hanoi using both plating and broth culture techniques. *E. coli* was chosen because of its suitability as a low cost indicator of fecal contamination of drinking water, with internationally accepted standards for collection and assessment (38). A biohazard awareness or training procedure for activities on farms was not required by either Canadian or Vietnamese universities. Biohazard training and procedures were in place for Vietnamese water testing facilities. Results were prepared using summary statistics and probit regression methods utilizing the software indicated above. The approach to fitting the probit regression models was a combination of plausibility of association, goodness-of-fit (pseudo-*R*^2^) measures, test of specification (Lagrange multiplier statistic), test for omitted variables (LR test), and concordance of correctly predicted outcomes (discriminant analysis).

## Results

Summary statistics of the demographic, economic, and some qualitative variables are shown in Table 1. We do not report summary statistics by province, which did not vary highly significantly between provinces. The most popular choice of crop grown was rice (441 farmers), followed by vegetables (293 farmers), and fruit (241 farmers). Many farmers grew at least two of these crops; for example, 441 farmers grew rice and vegetables and 199 farmers grew all three crops. All farmers reported they produced some kind of livestock and many produced fish (292 farmers). Livestock produced included chickens (476 farms), ducks (309 farms), pigs (304 farms), and cattle (198 farms). Less than half of farms (267) raised both pigs and chickens, and fewer still (139) raised pigs, chickens, and ducks on the same farm. The mean hours per day spent in contact with water (3.5) reflects time spent working with primarily fish ponds, but also reflects indirect contact with ducks and with water used in barns, often sourced from ponds or rivers.

**Table 1:** Basic summary statistics for small scale farmers in Vietnam (*n* = 598).

**Variable**	**N^1^**	**Mean**	**s.d**.	**Min**	**Max**
Age	597	45.94	11.31	17	85
Number of household members	598	4.40	1.34	2	10
Number of household members < 18 years	598	1.18	0.96	0	5
Years of schooling	598	6.89	3.23	0	18
Years farming	598	9.64	8.27	0	50
Hours per day in contact with water for agricultural production	567	3.48	5.21	0	20
Number chickens on farm^2^	571	26.03	30.84	1	500
Number ducks on farm	484	138.27	207.29	1	3,000
Number pigs on farm	401	22.84	24.52	1	200
Number cattle on farm	598	4.75	9.71	1	226
Training interest—farm mgmt.^3^	563	3.08	1.10	1	5
Training interest—water mgmt.	574	3.14	1.11	1	5
Has heard of *E. coli*	221	n/a	n/a	n/a	n/a
Has heard of avian influenza	541	n/a	n/a	n/a	n/a
Has heard of both *E. coli* and AI	215	n/a	n/a	n/a	n/a
Covers water storage	438	n/a	n/a	n/a	n/a
Treats water storage	570	n/a	n/a	n/a	n/a
Covers and treats water storage	419	n/a	n/a	n/a	n/a
Drinking water samples positive for coliforms (pres./absence)	456 of 560	n/a	n/a	n/a	n/a
Drinking water samples positive for coliforms (laboratory)^4^	466 of 582	n/a	n/a	n/a	n/a
Mean cfu *E. coli* in drinking water^5^	581	739.74	10,077.24	0	213,156

1 *Although 600 households were sampled, two households were dropped after data collection due to lack of animals and aquaculture*.

2 *Mean numbers of animals reported are for those who have any of each species*.

3 *For training interest, farmers were asked to rate their interest in management training ranging from 1 (no interest) to 5 (extremely interested)*.

4 *453 of 456 farms yielding a positive presence/absence test result for coliforms also yielded a positive result for E. coli in laboratory testing*.

5 *Based on laboratory test results*.

For their primary source of drinking water, 170 farmers reported they used well water while 119 reported they used rain water. Water from both sources is typically stored in a cistern of some kind, which is often but not always covered, and which may or may not be routinely treated or cleaned. When asked if they covered their water storage units, 438 (73.2%) respondents stated they covered their cisterns, while 160 (26.8%) did not. Similarly, when asked if they treated their drinking water cisterns (e.g., use of chlorine per FAO guidelines), 293 (49%) stated they did treat while 305 (51%) did not. Other sources of water used included piped, river, pond, or bought water.

The questionnaires and interviews also asked some general awareness questions relating to public health: 541 farmers said they had heard of highly pathogenic avian influenza (HPAI), 443 said they had heard of microorganisms in general, 221 had heard of *E. coli*, and 215 had heard of both HPAI and *E. coli*. Only 37 respondents said they would report sick birds to authorities if they were concerned the birds might be infected with HPAI. Reasons provided during semi-structured interviews and forums included lack of compensation, fear of prolonged loss of livelihood, and concern for reaction from neighbors. Several farmers cited cost or inconvenience as a barrier to engaging more thoroughly in all or most of the recommended procedures to prevent avian influenza on farm. In general, the qualitative analysis confirmed or provided further details to much of the quantitative findings, including opportunities to explain why farmers felt the way they did regarding, for example, motivation to protect wells from wild birds or why they would be less likely than their neighbor to get sick from avian influenza. Due to space limitations we do not provide extensive details of the qualitative analysis in this paper but findings have been prepared for a separate publication.

The probit regression models of Tables 2, 3 capture the significance of demographic, agricultural, and perceptions (e.g., perception that cost is a barrier to engaging in mitigating strategies; perception that the respondent is vulnerable to illness from HPAI) variables as predictors of the stated mitigating actions of farmers against waterborne disease either by protection of source or treatment of water. This approach attempts to identify predictors of behavior in both demography as well as beliefs. The former is consistent with policy analysis approaches in which policy formulators wish to identify characteristics of a likely successful behavioral change policy; the latter is consistent with the HBM.

**Table 2:** Probit regression results for Vietnamese small scale farmer engagement in mitigation of waterborne disease at source by biosecurity of source.

**Variable**	**Beta**	**s.d**.	**z-score**	***P* > |z|**
Years schooling	0.0892^**^	0.0384	2.32	0.020
Years farming	0.0188	0.0165	1.14	0.254
Household members >18	−0.0037	0.1230	−0.03	0.976
**RESPONDENT**				
Male health role	−0.1877	0.3336	−0.56	0.574
Female health role	0.9059^**^	0.3705	2.45	0.014
On-farm income	−0.3427	0.3494	−0.98	0.327
Chickens	−0.0016	0.0044	−0.35	0.723
Pigs	0.0161^**^	0.0076	2.12	0.034
Cattle	0.0028	0.0080	0.35	0.729
Log *E. coli* drinking water	−0.0608	0.0560	−1.09	0.277
**DUMMY WATER SOURCE**				
Rain	2.0403^***^	0.4991	4.09	0.000
Well	1.9960^***^	0.5143	3.88	0.000
Pipe	0.2796	0.5024	0.56	0.578
River with flocculation	0.2929	0.5158	0.57	0.570
**PERCEPTIONS**				
Susc to HPAI (agr water)	−0.1276	0.2446	−0.52	0.602
Susc to HPAI (well water)	0.7442^**^	0.3767	1.98	0.048
Severity HPAI	−0.7742	0.4885	−1.58	0.113
Cost is a barrier	−0.4789^*^	0.2498	−1.92	0.055
Knowledge barrier	0.1844	0.2632	0.70	0.483
Peers barrier	−1.0684^**^	0.4636	−2.30	0.021
Benefits encourage	0.5251	0.3620	1.45	0.147
Ability livestock	0.2463	0.2389	1.03	0.303
**TRIGGERS TO ACTION**				
Health worker advice	0.8031^***^	0.2774	2.90	0.004
Lost income	0.0082	0.3096	0.03	0.979
Peers	0.8845^***^	0.3314	2.67	0.008
Worry	0.4100	0.2706	1.52	0.130
Constant	−2.9400	0.7657	−3.84	N/A

*Dependent binary variable = Yes or No answer to the question “Indicate if you have been employing one or more of the following source water protection methods [with 9 options including “Cover drinking water reservoir, Human/animal waste treatment at source, Using buffer zones (e.g., vegetable garden), Other]”; Yes = 186, No = 118; Number of observations = 304; LR $\chi_{\left(26\right)}^{2}$ = 225.04; Prob > χ^2^ = 0.0000; Log likelihood = 95.0037; Pseudo R^2^ = 0.5422*;

* *P < 0.10*;

** *P < 0.05*;

*** P < 0.01; Concordance = 86.51%

**Table 3:** Probit regression results for Vietnamese small scale farmer engagement in mitigation of waterborne disease at source by treatment of water.

**Variable**	**Beta**	**s.d**.	**z-score**	***P* > |z|**
Years schooling	0.0632	0.0399	1.59	0.113
Years farming	0.0195	0.0169	1.16	0.247
Household members >18	0.0115	0.1300	0.09	0.929
**RESPONDENT**				
Male health role	0.1008	0.3474	0.29	0.772
Female health role	1.4816^***^	0.4397	3.37	0.001
On-farm income	−0.9672^***^	0.3344	−2.89	0.004
Chickens	−0.0008	0.0047	−0.16	0.871
Pigs	0.0172^*^	0.0106	1.63	0.103
Log *E. coli* drinking water	0.0181	0.0608	0.30	0.765
**DUMMY WATER SOURCE**				
Rain	1.5554^***^	0.5100	3.05	0.002
Pipe	0.2886	0.5032	0.57	0.566
Well	2.1583^***^	0.5893	3.66	0.000
River with flocculation	0.1225	0.4841	0.25	0.800
**PERCEPTIONS**				
Susc to HPAI (from agr water)	−0.3159	0.2602	−1.21	0.225
Susc to HPAI (from well water)	0.7093^*^	0.3845	1.84	0.065
Severity HPAI	0.6662	0.4682	1.42	0.155
Cost is a barrier	−0.6230^**^	0.2636	−2.36	0.018
Knowledge barrier	0.6054^**^	0.2798	2.16	0.031
Peers barrier	0.0639	0.5397	0.12	0.906
Benefits encourage	1.3906^***^	0.4408	3.15	0.002
Ability livestock	0.4928^*^	0.2757	1.79	0.074
**TRIGGERS TO ACTION**				
Health worker advice	0.8077^***^	0.2787	2.90	0.004
Lost income	0.6833^**^	0.3430	1.99	0.046
Peers	0.5982	0.3913	1.53	0.126
Worry	−0.1829	0.2958	−0.62	0.536
Constant	−4.3756	0.9117	−4.80	N/A

*Dependent binary variable = Yes or No answer to the question “Indicate if you have been employing one or more of the following mitigation practices in relation to water storage treatment [with 5 options including “Invest in household water treatment technology; Use and/or change disinfectants; Repair household water storage]”; Yes = 149, No = 155; Number of observations = 304; LR $\chi_{\left(25\right)}^{2}$ = 233.44; Prob > χ^2^ = 0.0000; Log likelihood = −84.3955; Pseudo R^2^ = 0.5804*;

* *P < 0.10*;

** *P < 0.05*;

*** P < 0.01; Concordance = 86.51%

The results of the probit regression for Vietnamese small scale farmer engagement in mitigation of waterborne disease at source by water biosecurity methods are shown in Table 2. The dependent variable, engagement in mitigation strategies at source, seeks to identify farmers who self-identify as engaging in several actions that protect drinking well water from contamination. This outcome variable was measured as a binary choice variable based on cumulative positive responses to nine mitigation options including covering the water storage unit, preventing livestock and wild bird access to wells, maintaining wells in good working condition, and preventing sewage runoff into wells. Household respondents had to indicate they used at least three of these offered measures to score a 1 (i.e., 1 = engages in mitigation strategies); otherwise, they scored zero.

The best-fit probit model contained 10 significant of 26 independent variables, including raising of pigs, years of schooling, using rain or bore well as a water source, and perception of susceptibility to HPAI from contaminated well water (all positively associated), and cost as a barrier to mitigation (negatively associated). The likelihood to engage in a health preventive action based on the advice of community health workers or peers were also significantly positively associated with the mitigation outcome variable. We were not able to show a positive association with the independent variables years of farming, raising chickens or cattle, or perception that lack of knowledge is a barrier to action.

The results of the probit regression for Vietnamese small scale farmer engagement in mitigation of waterborne disease by treatment and disinfection of water are shown in Table 3. The dependent variable, engagement in mitigation strategies by treatment, seeks to identify farmers who self-identify as engaging in several actions that treat drinking well water and/or storage facilities. Engagement in mitigation strategies was measured as a binary choice variable as described above, with the difference that options included storing water in confined tanks, investing in household water treatment technology (e.g., small scale filtration equipment), using disinfectants, and repairing water storage. As for the previous regression, respondents had to state they use at least three options to score a positive outcome. The same set of independent variables was used for both probit models. The best-fit probit model contained 12 significant of 25 independent variables, including raising of pigs, having a female in charge of family health decision making (both positively associated), and perception that cost is a barrier to engaging in mitigation (negatively associated). Self-declared perception of having good abilities in livestock management including manure management was positively associated with the outcome variable, as well as lost income as a trigger to action (i.e., if farmers knew with certainty income were lost due to not engaging in treatment of water, would they take action to change).

The following variables were significant (positively associated) for both models: having a female in charge of family health decision making, raising of pigs, having either rain water or a drilled well as primary water sources, perceived susceptibility to HPAI from well water, and the advice of a health care worker. Cost as a barrier to mitigation was significantly negatively associated for both models. Years of schooling was only significant for the biosecurity model specified in Table 2, but was commonly a significant predictor for several other specifications (not shown here) of both models. Concordance was more than 85% for both models; pseudo-*R*^2^ values and other test statistics can be seen at the bottom of both tables.

## Discussion

The presence of livestock on small scale mixed agriculture farms in Vietnam is known to be a source of potential infection from zoonotic disease and a concern for emerging infectious diseases (EIDs) in Southeast Asia (13, 39, 40). The VAC model has been successfully used for centuries in Vietnam as an ecologically balanced approach to profitable integrated agriculture and a useful method for recycling of biological waste. However, the emergence of HPAI in Southeast Asia challenged not only the VAC model but also the raising of poultry in Vietnam where chickens and ducks are not only a valuable source of protein and cash, but also important elements of the rice and fish cultivation systems for many farmers, small to large scale.

A common key factor of EIDs is water, illustrated clearly in Southeast Asia in recent emergences of HPAI (role of waterfowl in maintaining and transmitting H5N1 to domestic poultry), dengue (role in vector maintenance), and schistosomiasis. The role of livestock and water has been contentious but there is no question that raising of livestock in open management systems has contributed to the complicated picture of EIDs (41, 42). However, recognition of this risk factor and integrated management with water systems can have significant impact on reducing those risks (22, 43).

The VAC model in Vietnam has been a valuable tool for promoting sustainable integrated livestock and fish management while assuring a reliable source of household income, contributing to poverty alleviation for small scale farmers with limited alternate livelihood options. The VAC model follows classic recommendations of farm management that secure income and environmental stewardship including diversification of operations, recycling of animal waste in a non-toxic manner, and respect for the environment as a resource for future generations. Within this model, management of water is of course critical, and the emergence of water as a risk source of EIDs is an important concern that jeopardizes the future of the VAC model. Disruption of the model was considered by the Government of Vietnam during the early stages of HPAI emergence (25), although this was avoided by the use of vaccination as well as containment of ducks and separation from other poultry using netting (24, 44). Because of the challenges of maintaining biosecurity on small scale livestock farms including restricting access to pond water by migrating birds or other animals, the VAC model is likely to continue to face scrutiny.

Identifying actions and beliefs of farmers that are more likely to contribute to mitigating actions that are thought to reduce risk of EIDs (e.g., engage in on-farm biosecurity to protect water sources, and engage in treatment and maintenance of water storage vessels such as wells) is likely to reduce focus on water on small scale mixed agriculture farms as a source of EIDs. Because the VAC model is popular with such small scale farms, there is room to consider how identification of such actions and beliefs can be used to support rather than undermine the VAC model. We address this in our policy suggestions below.

It is interesting to note that those farmers in our study who were more likely to mitigate against waterborne disease had the following attributes that were significantly different predictors of their mitigating behavior than for their peers: raised pigs, expressed concern that they are more susceptible than average to HPAI from well water, a female family member is the decision maker for health decisions, recognize cost of health care as a barrier to health interventions, and follow the advice of health care workers. There are several interesting insights from these significant findings.

Pig manure is used to feed fish; it is simply pushed into the pond where it causes no harm to the fish and in fact they thrive on the additional nutrients it provides them. However, pig farmers are aware the manure is messy, bears a strong odor, and is not something they want tracked into the house. This perspective probably makes them more conscience of preventing contamination of water sources with pig manure and other contaminants. If individuals state they value the advice of health care workers and recognize health interventions are costly (i.e., value health care as a resource) then we can reasonably conclude they likely value this knowledge and resource enough to take the effort to follow through on extended actions including mitigating actions against waterborne disease.

The significance of well and rain water sources compared with other sources of water as a predictor of mitigation against waterborne disease is also highly intriguing. We suggest there are several reasons for this. Those farmers who take the time and effort to catch rain water and manage a bored well are more likely to be concerned with the quality and cleanliness of that water. Those farmers who use river or piped water are more inclined to view it as a resource that requires little maintenance (i.e., it always seems to be there when they need it), and thus perhaps pay less attention to the quality of the water at source. It should be noted that most of the farmers in our study area do claim that they filter their water (results not shown here), so we are not claiming they are not concerned about water quality, only that piped and river water users seem less concerned with protecting the water at source.

Our general observation that many of the small scale farmers owning livestock in our study do perceive mitigation against contamination of water important to maintaining good health is consistent with findings from other studies. Previous research has shown that agricultural producers including poultry farmers identify animal waste management as important to preventing disease (45, 46), particularly where there is contact with water associated with agricultural production (16, 19, 20). Interestingly, we also found consistency in previous research with the perception of many of our respondents that HPAI cannot happen to them (27, 45). Although there was some evidence in previous research that some strata of small scale farmers do not mitigate against EIDs, this seems to be associated with a result of lack of risk awareness (16, 18).

We used the HBM as a theoretical underpinning of our hypothesis that there is a strong association between Vietnamese small scale farmers' perception of risk of disease of waterborne origin and engagement in mitigating actions related to protection of water sources from contamination. We were able to show consistently in both our models that engagement in mitigating actions is associated with perceptions of susceptibility to disease from HPAI, belief that cost is a barrier to taking action, and health worker advice is a trigger to action (all specific examples of pillars of belief in the HBM). Other explanatory variables describing perceptions were also significant for either but not both models. Thus we have demonstrated to a modest extent that the HBM is a suitable choice for explaining mitigating behavior of small scale poultry farmers in Vietnam. Nevertheless, we expected to find a stronger association of several predictive variables including knowledge as a barrier in both models and potential lost income as a cue to action. This may be an artifact of econometric specification, a result of poorly worded question and thus weak data, or indeed an indication that potential for lost income is not consistently a motivating factor. These considerations suggest areas for further research.

While this research focused on *E. coli* and avian influenza as diseases of concern on small scale mixed farms in Vietnam, we propose broader policy recommendations aimed at reducing the risk of EIDs on small scale mixed farms including those using the VAC model. The preliminary integrated national plans for controlling HPAI in Vietnam (25, 44) focused on vaccination, improving the capacity of animal and human health services to detect and respond, and minimizing economic losses for small scale farmers. Actionable mitigation plans included very general suggestions for animal waste management and protection of water sources. However, there has not been a detailed outline of possible mitigating actions addressing water and animal waste management presented to small scale farmers that has been supported by extension education and value recognition.

We propose agricultural extension policy that supports such a programme, which should include checklists for mitigating actions, farmer field training to increase engagement, regular testing of on-farm water for presence of waterborne zoonotic pathogens of highest concern, and recognition of achievement. For example, establishing Standard Operating Procedures (SOP) with tiered certification achievement could be tied to awareness and information campaigns in local markets to promote premiums paid for agricultural products produced from SOP certified farms. Further research should also address the deeper motivating factors behind mitigating behavior change (the “why” of change), and how that can be tied to on-farm risk awareness. Our results provide a starting point for identifying small scale farmers who do engage in such mitigating actions although we feel stronger evidence and wider national studies are needed.

There are some weaknesses of our research. Although we feel our sample size was adequate for the strength or results stated and that Thai Binh and An Giang are highly representative of provinces in Vietnam with high numbers of small scale farmers, we are not able to generalize our results for all small scale mixed farmers in Vietnam. Although small scale chicken and duck production is highly similar, water sources vary across provinces, as does the economic importance of fish and cattle to household income. Experiences with HPAI in both poultry and humans have varied as well, with resulting impact on depopulation on poultry farms and probably different perceptions as to the risk of EIDs. As well, we did not account for seasonal change, or stratification of economic contribution from various on-farm activities.

## Conclusions

Our research identifies several attributes and characteristics of farmers and integrated small scale farms in Vietnam that can be used in a policy framework to sustain the economic and environmental benefits of the VAC model while reducing the risk of EIDs from water contaminated with livestock waste. Water and farm management policy options to support agricultural economic activity in rural areas while promoting sustainable eco-friendly methods targeting low income small scale farmers would benefit from addressing mitigation strategies against waterborne disease. Such policy options should consider the livestock species raised, source of water (rain and well users are more likely to benefit), and awareness of susceptibility to waterborne disease. Improving the latter and increasing access to health care advice as well as promoting well and rain water as sources of water are also elements of what we see as a likely successful water and farm management policy in Vietnam.

The VAC model should continue to be promoted in Vietnam due to the ecological and poverty alleviation benefits. However, there is a need to address the increased risk of waterborne disease inherent with small scale mixed farming in general. We have identified several factors and characteristics of small scale farmers in Vietnam that we see as valuable to water and farm policy development, as well as brief suggestions for a rural agricultural policy incorporating certification of SOPs to support increased adoption of actions on small scale farms to mitigate against EIDs.

## Author contributions

DH was the Principal Investigator on the project, secured the grant, and led the analysis and writing of the manuscript. DH and QL designed the study. QL was a doctoral student researcher on the project, led the data gathering for the project, and assisted with the analysis and writing of the manuscript.

### Conflict of interest statement

The authors declare that the research was conducted in the absence of any commercial or financial relationships that could be construed as a potential conflict of interest.

## Footnotes

^1^In order to achieve statistically significant results at a margin of error of 5%, sample size was calculated using a standard formula for normally distributed data (35) as: sample size = S = [(z^2^p(1-p))/e^2^]/[1+((z^2^p(1-p))/e^2^N] where *N* = population size (i.e., c12,000 per province), e = margin of error = 5%, *p* = proportion with variable of interest (i.e., keep poultry, fish, and crops) = c.0.75, and z = z-score = 1.96 for 95% confidence interval. Thus, S = [(1.96^2^*0.75*0.25)/0.05^2^)/(1+((1.96^2^*0.75*0.25))/0.05^2^*12,000] = 281 per province.
